# Research and the meaning of ‘public interest’ in POPIA

**DOI:** 10.17159/sajs.2022/13206

**Published:** 2022-03-07

**Authors:** Donrich Thaldar

**Affiliations:** 1 School of Law, University of KwaZulu-Natal, Durban, South Africa

**Keywords:** Code of Conduct for Research, definition, POPIA, public interest, South Africa

The term ‘public interest’ is used 16 times in the *Protection of Personal Information Act 4 of 2013* (POPIA). Understanding the concept is important for research. This is because the public interest is relevant when considering a research exception for allowing the processing of special personal information (section 27(1)(d)(i)), and a research exemption from the conditions for processing personal information (section 37(1)(a)). But what exactly does public interest mean?

In the absence of a definition in POPIA itself, the Information Regulator proffered the following ‘basic formulation’ of public interest in a recent Guidance Note^1^:

Public interest is a wide and diverse concept that cannot, and should not, be limited in its scope and application. The definition of what constitutes public interest varies across jurisdictions and should be assessed on a case-by-case basis. In its very basic formulation public interest is the notion that an action or process or outcome widely and generally benefits the public at large (as opposed to a few or a single entity or person) and should be accepted or pursued in the spirit of equality and justice.

As I show here, this basic formulation of public interest is misaligned with the way in which public interest has been interpreted by South African courts and hence is in need of revision. Furthermore, it would assist the research community if the basic formulation could be expanded by the Code of Conduct for Research (the Code) that the Academy of Science of South Africa (ASSAf) is currently developing.^2^ To inform ASSAf’s Code development process, this article provides a concise analysis of the principles concerning public interest that have crystallised in our law.

In specific contexts, such as where it forms part of the statutory mandate of a public body, the meaning of public interest may become complex and contentious, as illustrated by the *Rail Commuter Action Group* series of cases.^3–5^ However, in this article I confine the analysis to general principles relevant to research in the context of POPIA.

## The basic formulation reconsidered

Similar to the Information Regulator’s Guidance Note, the South African courts have called public interest a ‘broad and uncertain’^6^ concept that does not permit a clear, precise and comprehensive definition.^6–8^ Yet a good general understanding of the concept can be developed by considering the ways in which the courts have interpreted and applied the concept. At a general level, public interest has been interpreted as benefiting the public^4,8^, promoting the general welfare of the public^3^, or better serving the public^6,7^. Although there may be differences in nuance between these choices of wording, for present purposes they effectively have the same meaning. Accordingly, the Information Regulator’s use of the word ‘benefit’ in its basic formulation of public interest is appropriate and aligned with our law.

However, this is where the alignment between the Information Regulator’s understanding of public interest and the actual meaning of public interest as expounded by South African courts stops. Importantly, the Information Regulator’s interpretation of the ‘public’ as meaning the ‘public at large’ is not necessarily true. The courts have made it clear that the ‘public’ concerned need *not* be widely representative of the general public – it can be a smaller community within the broader national community.^6,8,9^

In this context it is also important to note that the public interest does not depend on each member of the relevant community or ‘public’ directly benefiting. As succinctly held by the High Court^10^:

[A] scheme is ‘in the public interest’ if it is to the general interest of the community that it should be carried out, even if it directly benefits only a section or class or portion of the community.

While private interests and the public interest can overlap (or be in conflict), the public interest is not merely an aggregate of private interests. The word ‘general’ in the quote above indicates that something broader than the private interests of individuals is envisaged.^7^ Accordingly, although direct benefits may befall only a section of the community, the relevant consideration is whether the community (the relevant ‘public’) would *qua* collective reap (indirect) benefit. This can be illustrated by three examples from case law: (1) introducing competition in a regulated market (rather than a monopoly) in Mookgopong (previously Naboomspruit) was held to be in the public interest^8^, even though there is direct benefit for the individual new competitor that is granted a licence; (2) a rebuilding scheme to answer the demand among persons of a certain profession for office space in downtown Johannesburg was held to be *prima facie* in the public interest^10^, even though there is direct benefit for the individual landlord; and (3) the principle of open justice (court proceedings being open to the public, rather than held in secret) was held to be in the public interest^11^, even though there is direct benefit for the media outlets that are reporting on the cases. It is worth noting that while the relevant ‘public’ in example (3) was the South African public at large, in example (1) the ‘public’ was a small town, and in example (2) it was a class of persons defined by their common profession.

From examples (1) and (2) it should also be clear that ‘the spirit of equality and justice’ is a novel criterion invented by the Information Regulator without any basis in law. Although equality and justice are certainly core values of our common law and our constitutional dispensation, these values are simply not criteria for public interest. The focus on these two values also raises the question: Why elevate equality and justice above other constitutional values such as openness and individual freedom? While something that undermines equality or justice (or openness, or freedom, *et cetera*) will likely not be in the public interest, as illustrated by examples (1) and (2), there are many examples of things that the courts held to be in the public interest that do not actively promote or protect any of these values.

Given my analysis above, I suggest that the basic formulation for being in the public interest ought to be whether something *benefits the people of South Africa qua collective, or benefits a group of people in South Africa qua collective*. Such benefit may be indirect, and any direct benefit to one or more individuals does not detract from the indirect benefit to the relevant public. Also, benefit to the relevant public is sufficient to constitute public interest; it need not be shown that ‘the spirit of equality and justice’ or any other value of our law is being promoted or protected.

## Application

I now consider the application of the revised basic formulation of public interest to exceptions and exemptions for research in terms of POPIA.

Section 27(1)(d)(i) (exceptions to the prohibition on processing of special personal information) entails a simple public interest test: either the research study in question serves the public interest or not. Accordingly, the revised basic formulation should be used as a general guideline. It would further help the research community if examples of research studies that would be deemed to be in the public interest could be provided by the Code. It may also be good if the Code makes it clear that the public interest requirement need not be satisfied by the research study itself, but may be satisfied by the envisioned application of the study’s results, the resource investment made during the study, the employment created, or any other downstream benefits.

Health research is a special case.^12^ Consider that all health research must be approved by a health research ethics committee and that these committees have a statutory duty to ensure that health research will ‘promote health, contribute to the prevention of communicable or non-communicable diseases or disability or result in cures for communicable or non-communicable diseases’^13^. The outcome is that *all* health research conducted in South Africa is *prima facie* in the public interest. To deny this would amount to a denial that the system of ethics review in South Africa is robust and as a general rule functions well. Although there have been some exceptions, there is no evidence in the public domain to cast general doubt on the system of ethics review in South Africa.

In contrast with section 27(1)(d)(i), section 37(1)(a) (exemptions from the conditions for processing of personal information) assumes that research is in the public interest (see section 37(2)(e)) and focuses on the importance of the public interest served. This implies a potential hierarchy among different instances of the public interest. The idea that some instances of the public interest (or simply ‘public interests’) may be more important than others is also found in case law. For example, the court held, with reference to English case law, that one of two competing public interests is of a ‘high order’.^14^ Section 37(1)(a) requires that ‘the public interest in the processing *outweighs, to a substantial degree*, any interference with the privacy of the data subject that could result from such processing’ (my emphasis). Accordingly, while some instances of the public interest may substantially outweigh the data subject’s privacy rights, some may not. Clearly, section 37(1)(a) entails a *balancing exercise*, where the inevitable question is how to allocate weight to the conflicting interests.

I suggest that this balancing exercise should be informed by the Constitution *qua* the ultimate normative guide in South African law.^15^ The Code can play an important role in facilitating the balancing exercise by applying constitutional rights and values to the *research* context and identifying factors that should be considered. Given that the Code aims to be inclusive of all academic disciplines, it should provide for a diverse range of factors, which gives researchers from all disciplines a fair opportunity to demonstrate the importance of their intended research.

It bears repetition that equality and justice – important as they are – are not the only constitutional values, and that values such as openness, dignity, freedom, and *ubuntu* should not be sidelined.

## Concluding note

The guidance provided by the Information Regulator should be well informed and aligned with the law of the land. Although the Information Regulator has wide-ranging powers, it cannot create law. Similarly, it cannot limit the rights bestowed by POPIA through a restrictive interpretation of the meaning of public interest. Members of the South African research community have the right, where relevant, to seek a research exception to allow the processing of special personal information (section 27(1)(d)(i)), and to seek a research exemption from the conditions for processing personal information (section 37(1)(a)) provided for in POPIA. When exercising this right, the research institution (or individual researcher) is entitled to rely on the broader, more inclusive meaning of public interest that is found in South African law, rather than on the description of public interest provided by the Information Regulator in its Guidance Note.^1^ Neither the Guidance Note nor the Code can override the judicial view of what public interest is. At best, these documents can explain and elaborate on the judicial view of public opinion. To avoid confusion, the Guidance Note should be amended in line with the revised basic formulation of being in the public interest that was developed in this article.

Furthermore, this article provides basic pointers regarding the application of public interest to research in the context of POPIA. Building on these pointers, the Code should, at a minimum: (1) distinguish between instances of research that are in the public interest from those that are not; and (2) where research is deemed to be in the public interest, allocate weight to the public interest being served and the privacy interest that is to be limited. Both (1) and (2) should be done in a meaningful, predictable, and reproducible manner.

## Significance:

‘Public interest’ is an important concept in POPIA. However, the way in which it has been interpreted by the Information Regulator is subject to criticism. A better interpretation is suggested.
